# Participation of Leukotrienes in the Immune Modulation of Oral Tolerance

**DOI:** 10.3389/fmicb.2017.00242

**Published:** 2017-02-21

**Authors:** Sandra R. P. de Oliveira, Auro Nomizo, Fabiani G. Frantz, Lúcia H. Faccioli, Ana Paula Keller de Matos, Emanuel Carrilho, Ana Afonso, Fernanda de Freitas Anibal

**Affiliations:** ^1^Laboratory of Parasitology, Department of Morphology and Pathology, Universidade Federal de São Carlos São Carlos, Brazil; ^2^Faculdade de Ciências Farmacêuticas de Ribeirão Preto – University of São Paulo Ribeirão Preto, Brazil; ^3^Universidade de São Paulo, Escola de Enfermagem de Ribeirão Preto, Ribeirão Preto Brazil; ^4^Bioanalytical, Microfabrication, and Separations Group, Instituto de Química de São Carlos, Universidade de São Paulo São Carlos, Brazil; ^5^Medical Parasitology Unit, Global Health and Tropical Medicine, Instituto de Higiene e Medicina Tropical, Universidade Nova de Lisboa Lisbon, Portugal

**Keywords:** oral tolerance, leukotriene, cytokines, immune response, IL-5, IFN-γ

## Abstract

Oral tolerance (OT) is characterized as a peripheral immune tolerance form, in which, mature lymphocytes in lymphoid tissues associated with mucosa, become non-functional or hypo responsive due to prior oral administration of antigen. OT is an important immunological phenomenon due to its therapeutic potential in inflammatory processes and others diseases. Here we evaluated leukotriene role in the induction of OT, as well as, the production of cytokines IL-5 and IFN-γ in leukotriene deficient animals (knock-out). Our results suggested that even in the presence of OT and leukotrienes absence, cytokine IFN-γ remains being secreted, which gives us an indication of immune system specificity and also that IFN-γ participates in various immune processes.

## Introduction

Previous work from other authors brings oral tolerance (OT) not only as a new tool for studying new therapies for auto-immune processes control but also to reduce chronicity and regulate all inflammatory responses, especially in the pathophysiology of allergic diseases (Faria and Weiner, 1999; Mayer and Shao, 2004). Mechanisms that regulate the immune response have been proposed to explain OT, and it is known that leukotrienes are of great importance in modulating all inflammatory processes, especially those that are characterized by an immune response profile of T helper type 2 T (Th2). Additionally the triggered response in OT can regulate important cells such as regulatory CD4 + T cells secreting interleukin-10 (IL-10) or transforming growth factor β (TGF-β) and it may participate in the control of various cells, such as T cell helper type 1 (Th1) immune response (Piccirillo et al., 2002; Faria and Weiner, 2006). Further, profile-Th2 cells participate in mediating specific or non-specific suppression of antigen reactive cells (Miller et al., 1991; Silva et al., 2010). Thus, in the murine model of OT when we use low doses of antigen the elimination of regulatory cells in upregulated, whereas higher doses favor clonal expansion in anergy or deletion processes (Friedman and Weiner, 1994). Therefore, the murine model was chosen to evaluate the role of leukotrienes in the setting of OT.

Immunological tolerance is described as an active process in which T lymphocytes have a central function, immunological tolerance is recognized as an important regulation process of immune responses (Bluestone, 2011). Therefore, OT is characterized as a peripheral tolerance form, in which, mature lymphocytes in lymphoid tissues associated with mucosa become non-functional or hypo responsive due to the prior oral administration of antigen (Krause et al., 2000; Weiner et al., 2011).

The induction of local tolerance becomes important since it guarantees a state of low immune response to certain antigens administered in an immunogenic form, when they come in contact with other mucous membranes (Bluestone, 2011; Sgarbi and Mucida, 2012).

Oral tolerance does not develop, however, in situations where antigens persist and replicate in the intestinal mucosa, or in situations of pathogenic viruses and bacteria that invade directly this environment (Faria and Weiner, 2005).

Antigen administration through oral route for tolerization base itself on the fact that gastrointestinal tract is in close contact with a great variety of food, bacterial, parasites and chemical products, so to overcome this constant antigenic stimulation a negative regularization on systemic immune responses is necessary (Olivier, 2004; Gallegos and Bevan, 2006; Ramos et al., 2008). Such negative regularization on systemic immune responses becomes necessary, since the differentiation in the type of response is very important, to prevent responses against food antigens or edible bacteria, which might lead to food allergies, mucosa destruction or inflammatory disorders (Raff and Levitzky, 2012).

However, the induction and maintenance of an OT immune state are factors highly dependent on the antigen nature and dose, the frequency and interval of antigen exposition, the time gap between each antigen exposure, previous immunological experience, age when the first contact with the antigen occurred and other genetic factors (Krause et al., 2000; Raff and Levitzky, 2012). Establishment of OT makes use of specific antigen cells from peripheral lymphoid organs presenting reduction in the production of interleukins such as IL-2, IL-3, IL-4, IL-5, IL-10, and IFN-γ *in vitro*, and further, this response is accompanied by the inhibition of humoral response. (Chehade and Mayer, 2005).

Leukotrienes are produced by the metabolism of arachidonic acid (AA) by 5-lipoxygenase path-way, being secreted by various cells, such as macrophages, neutrophils, eosinophils, and mast cells (Bousquet et al., 2008). Conversion of AA to leukotrienes involves the participation of 5-lipoxygenase and FLAP protein (5-LO–activating protein), which converts AA to leukotrienes in an unstable form, LTA4 (leukotriene A4) is hydrolysed forming the LTB4 (leukotriene B_4_) or conjugated to three amino acids to form LTC_4_ (leukotriene C_4_). These reactions are catalyzed by LTA4 hydrolase. LTC4 is converted into LTD4 (leukotriene D4), and LTE4 (leukotriene E4) by extracellular metabolism and these three molecules are collectively referred as cysteinyl-leukotrienes (LTs *cis*) or peptide-leukotrienes (Singh et al., 2010; Nakamura and Shimizu, 2011). Lymphocytes express leukotrienes receptors, LTB4 is chemotactic for CD8+ T lymphocytes; cysteinyl-leukotrienes induce production of chemokine’s RANTES, (regulated upon activation, normal T cell expressed and secreted) which is an evidence that chemokine’s might be involved in OT. The immunosuppression is accompanied by immunological changes to maintain both peripheral tolerance and OT thus allowing to maintain immunological competence. Leukotrienes are potent 5-lipoxygenase-derived inflammatory mediators and characteristic of leukotriene-related diseases (e.g., hypersensitivities, asthma, and allergic rhinitis). Therefore, OT is a model used to understand the mechanisms and mediators during immunological anergy, in this study we evaluated the leukotriene role in the induction of OT, as well as the production of the cytokines IL-5 and IFN-γ in leukotriene deficient animals.

In this context, the aim of this study was to evaluate the participation of leukotrienes and modulation of cytokines IL-5 and IFN-γ during OT in leukotrienes (5-LO-KO) deficient animals.

## Materials and Methods

### Animals

Young females pathogen free mice belonging to the 129 and 5-LO-KO strains were used (animals deficient in 5-LO) both weighing between 18–22 g, all kept in the animal house of the Faculdade de Ciências Farmacêuticas de Ribeirão Preto – USP, with free access to water and food. This study was approved and conducted in accordance with the guidelines established by the University of São Paulo Animal Care Committee, n^o^ 02.1.1408.53.8.

### Oral Tolerance Induction and Immunization Protocol

Oral Tolerance was induced by oral gavage using syringe and metal cannula. It was administered 10 mg of Ova – grade III (Sigma, St. Louis, MO, USA) diluted in 200 μl of sterile PBS per animal for 3 consecutive days (groups TOL TOL and MK +). Control and immune groups received PBS during those 3 days. Seven days after the first dose of oral OVA, mice were immunized with 100 μg of OVA adsorbed in 1 mg of aluminum hydroxide and 200 μl of PBS/animal, intraperitoneally. Thirty days after the beginning of the experiment, a second dose of aluminum hydroxide and 200 μl of PBS/animal were administered, intraperitoneally. Control group received only PBS intraperitoneally during immunization and challenge. Twenty-eight days after OT induction, the animals were euthanized with an overdose of anesthetic.

### Evaluation of Immune Response

#### Analysis of Mesenteric Lymph Node Cells

After 30 days of treatment, the lymph nodes were removed and macerated. Cell suspensions were washed and re-suspended in complete RPMI-1640 (Sigma; supplemented with 5% of fetal bovine serum, 40 μg/ml of gentamicin, 20 mM Hepes).

In these tests, preparations of whole cell from organs were used. Cell suspensions containing 5 × 10^6^ cells in 200 μl were incubated in the presence or absence of OVA grade III – Sigma (200 μg/ml) or anti-CD3 (10 μl/ml) in 96 well, flat bottom (Costar 3595, Coming, NY, USA). Cells were cultured for 72 h at 37°C in an oven containing 5% CO_2_, and 18 h before the end of the culture, cells were incubated with [^3^ H] thymidine at a concentration of 0.25 μCi/well. Cells were collected in cells collector (Inotech cell Harvester, USA). Reading was held in a β-scintillation counter (Beckman, Brea, CA, USA). Posteriorly, the supernatants were collected and frozen at 20°C for subsequent measurement of the cytokines IL-5 and IFN-γ by ELISA.

#### Quantification of Cytokines IL-5 and IFN-γ Levels Serum in Culture Supernatants

ELISA was performed according to the manufacturer instructions (BD) using purified mAb as capture purified anti-mouse Abs (lot.5098896 to IL-5 detection; lot. 83480 to IFN-γ, both from BD Biosciences) to serum cytokines from samples, and biotinylated anti-mouse mAb (lot. 5098897 to IL-5 detection; lot.83479 to IFN-γ, both from BD Biosciences) for as cytokines detection, followed by incubation with streptavidin-peroxidase (HRP) and substrate TMP (BD Biosciences). The ELISAs for cytokines detection were performed according to the manufacturer’s instructions using commercial BD kits (standard curve) for IL-5 (15.6–1000 pg/ml, assay range) and IFN- _ (9.4–600 pg/ml). Plates were read in a 96-well spectrophotometer (Microquant-Sellex, Inc 450 nm) and data was analyzed using software by comparison against a standard curve generated using recombinant cytokine at known concentration (Montuschi, 2008).

### Statistical Analysis

The results were expressed as mean ± SEM. The results obtained in different experiments were analyzed using both ANOVA and Mann–Whitney. Statistical analysis was performed using PRISM (San Diego, CA, USA). The level of significance was 5%.

## Results

### Analysis of the Total Number of Cells in the Mesenteric Lymph Nodes of Animals 129 and 5-LO-KO after Induction of Oral Tolerance (TOL) or Immunization with OVA

**FIGURE 1:**
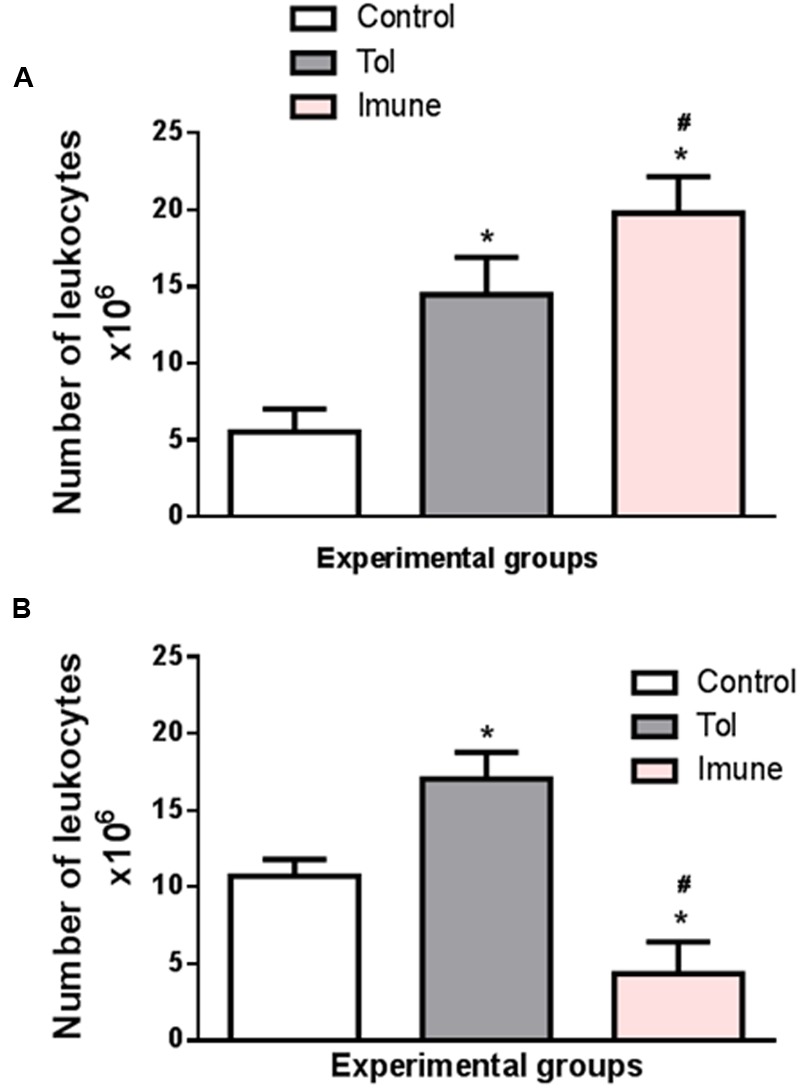
**The graph shows the total number of cells in the mesenteric lymph nodes of animals from the line 129 (A)** and 5 LO-KO **(B)** after induction of TOL and i.p. immunization, both with OVA. Data represent mean ± SEM (*n* = 6 animals). The statistical analysis of the cell proliferation was performed using the Tukey’s multiple comparison test with significance set at ^∗^*p* < 0.05 when the experimental group was compared to control group and ^#^*p* < 0.05 when compared TOL and immune.

Cell proliferation in the lymph nodes is essential to generate an immune response. **Figure 1A** shows the total number of cells in the mesenteric lymph nodes of animals from line 129, after induction of TOL and i.p. immunization with OVA. When compared to the control group, which only received PBS, the immune animals 129 (OVA/Alumen) and TOL (OVA) showed an increase in the total cell quantity (Montuschi, 2010).

The data presented demonstrate that the total number of cells (leukocytes) present in the mesenteric lymph nodes present significant differences between the different animals studied. Animals with wild type background (129) exhibit a larger number of cells in both TOL animals and those previously immunized with OVA. However, animals presenting background 5-LO-KO and therefore do not produce leukotrienes have a significantly lower number in the animals that were immunized with OVA, when compared to the other TOL groups and even the control animals. We found that animals immunized with OVA/Alumen (Immune) showed higher numbers in cell proliferation when compared to TOL animals. **Figure 1B** shows that the total cell proliferation in mesenteric lymph nodes is lower in these immunized animals when compared with the control and TOL groups (**Figure 1B**). However, when observing immune deficient leukotrienes animals, a significant decrease in cell number was observed.

### Analysis of the Modulation of IL-5 in the Mesenteric Lymph Nodes of Animals 129 and 5-LO-KO

**FIGURE 2:**
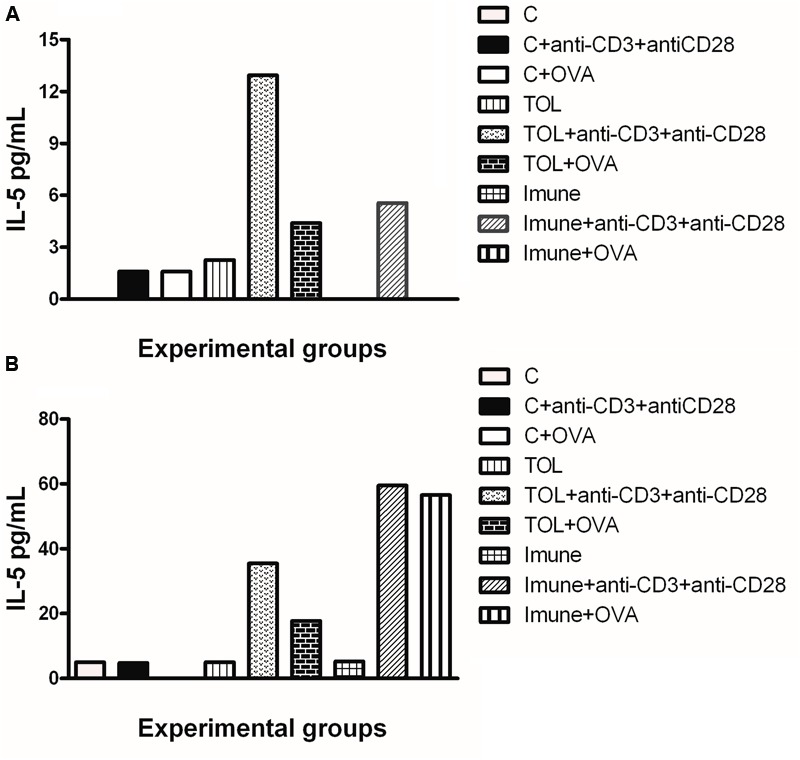
**Analysis of the concentration of IL-5 in the mesenteric lymph node of animals 129 (A)** and 5-LO-KO **(B)**. Data represent mean ± SEM (*n* = 6 animals). A comparison was made between treatments in culture, that is, between the cytokine basal production and the one stimulated with OVA.

Results in **Figure 2A** shows that mesenteric lymph node cells from animals 129 tolerized+anti CD3+anti CD 28 presented release of IL-5 more significant when compared to the other stimuli in these lines (**Figure 2A**). **Figure 2B** shows the levels of IL-5 in leukotrienes deficient animals (5-LO-KO). We found that the TOL group in the presence of antibodies (anti-CD3 and anti-CD28) induced cytokine production significantly. However, this same group when in contact with OVA and/or anti-CD3 and anti-CD28 stimuli, increased cytokine production on a discrete level.

### Analysis in the Modulation of IFN-γ in Mesenteric Lymph Node of Animals 129 and 5-LO-KO

**FIGURE 3:**
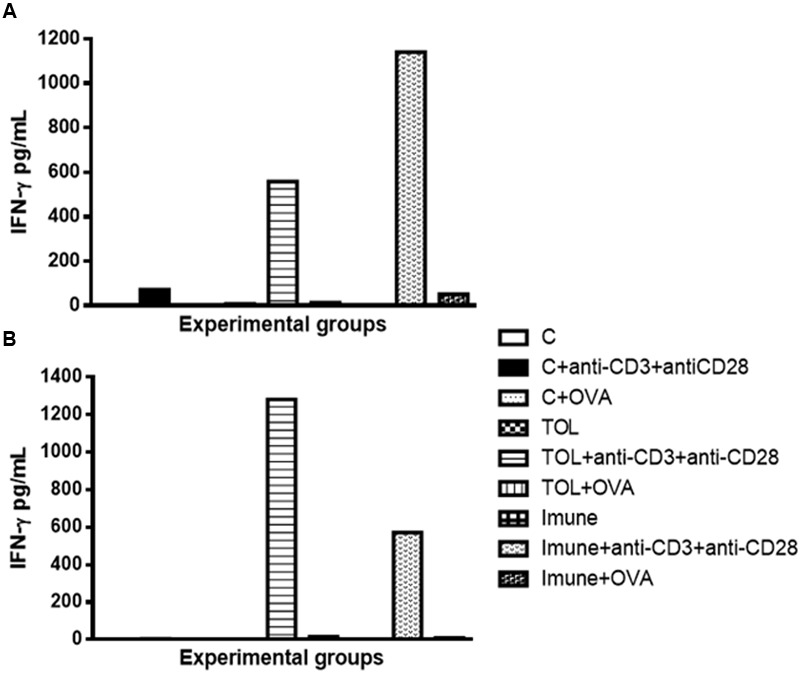
**Analysis of the concentration of IFN-γ in the mesenteric lymph node of animals 129 (A)** and 5-LO-KO **(B)**. Data represent mean ± SEM (*n* = 6 animals). The non-parametric Mann–Whitney test was used to study cytokine significance set at ^∗^*p* < 0.05. A comparison was made between treatments in culture, that is, between the cytokine basal production and the one stimulated with OVA.

Our results strongly suggested that the presence of anti-CD3 and anti CD28 stimulation, cells of immune animal 129, presented an increase in IFN-γ (**Figure 3A**), whereas, animals TOL on the same stimulus, showed moderate levels of IFN-γ (**Figure 3A**). Data presented in **Figure 3B** demonstrated a significant increase in IFN-γ production in animals 5-LO KO on experimental group TOL when in contact with anti-CD3 and anti-CD28 antibody.

## Discussion

Oral tolerance has been classically defined as a decrease in humoral and/or cellular reactivity after immunization with an antigen previously administered orally (Weiner et al., 1994; Smith and Nagler-Anderson, 2005; Round and Mazmanian, 2009). The inflammatory disease of the lungs and Th2-type cells that it’s released of IL-4 and IL5 play a very important role in its physio-pathogenesis. Chung et al. (2002) demonstrated that OT can be induced and maintained in a Th2-related immune response, and that an ongoing immune response can be suppressed by the oral administration of antigen combined with an appropriate feeding regimen. Nevertheless, the immunological events resulting from the oral administration of antigen combined with an appropriate feeding regimen are not fully understood.

We determined the total proliferative capacity of cells in the mesenteric lymph nodes (which represent one of the different routes of immune system circulatory migration) in culture and in the presence or absence of the antigen. Relating the cell proliferation rate of the control group, which received PBS via i.p. and water during periods of immunization and challenge, immune animals 129 (OVA/Alumen) and TOL (OVA) showed an increase in the number of total cells, as other authors showed (Macpherson and Smith, 2006).

We also found that animals immunized with OVA/Alumen (Immune) have higher rates of cell proliferation. Moreover, prior administration of the antigen orally (TOL) leads to the reduction in cell production when compared to the cellular immune group suggesting a slight suppression of cellular immune activity in these animals (Graph-1-A), according with Worbs et al. (2006).

Animals from the strain 5-LO KO are leukotrienes deficient because they do not catalyze the enzyme 5-LO. In our results we found that the total cell proliferation in mesenteric lymph nodes is low in these animals when they are immunized, compared to control groups and TOL (Graph 1-B). This might give an indication that the deficiency of leukotrienes, did not prevent the establishment of tolerance, but when we observed these animals leukotrienes immune deficient, we observed a significant decrease in cell number. Fierro et al. (2002) demonstrated a role in cell proliferation and migration dependent of leukotrienes, corroborant with our results Fierro et al. (2002). Therefore, these results suggest that leukotrienes are important mediators involved in specific immune response, for its absence partially prevented the immune response (Holgate et al., 2003).

Moreover, our data also corroborates with those described by Frantz (2004) where animals that have leukotriene synthesis inhibited by medication also have a low concentration of total antibodies, which may indicate a tolerance induction. Therefore, the absences of leukotrienes do not prevent the establishment of tolerance. Once OT is induced, various events of the reactivity immune/antigen specific are suppressed, and the production of immunoglobulin (Ig) classes such as IgE, IgG, IgM and IgA, as well as production of various cytokines (Weiner et al., 2011).

Concerning the production of IL-5, we found that in animals 129 the release of IL-5 was significant in tolerized animals + anti CD3 + anti CD 28 when compared to the other groups. These isolated results needed to be compared with the deficient strain of leukotrienes, so we can understand better from these mediators in OT and its relationship with IL-5. In other experimental models some authors shown that depending on the mice strain IL-5 and leukotrienes may participate in the inflammatory process independently (Anibal et al., 2007). However, we observed that IL-5 production in animals 5-LO-KO in the immune group increased significantly, suggesting that IL-5 in animals immunized with OVA operates in a manner partly independent of leukotrienes. Therefore, we suggest that IL-5 is more important than leukotrienes when animals are immunized than when they are in peripheral tolerance (Graph 2-B). These data differ from the results obtained in wild animals (129), in which we observed that IL-5 is more important in tolerance than in immunization (Graph 2-A). Moreover, Shimizu et al. (2013) showed that eosinophils, Th2 cytokines, and leukotrienes are involved in the physiopathology of this asthma model. It is known that IFN-γ is a cytokine present in several immune responses; we studied the release of the same in animals’ leukotriene deficient in the TOL model. Our data strongly suggests that during tolerance, leukotriene do not participate in the release of IFN-γ (Graph 3-B), as suggested by Possa et al. (2014), as for the immune response, when the stimulus was immunization, we observed the opposite, the absence of leukotrienes prevents the release of this cytokine (Graph 3-B). These data differ from the results obtained in wild animals (129), where we observed that IL-5 is more important in tolerance than in immunization (**Figure 2A**).

According to these results we can suggest that leukotrienes may be important in the regulation of OT once, which are involved in the modulation of cytokines evaluated in this study.

The role of leukotrienes in tolerance might be studied *in vivo* by measuring these compounds in various biological matrices including exhaled breath condensate (Montuschi et al., 2014).

So we can conclude that even in the tolerance and in the leukotrienes absence, the cytokines IFN-γ and IL-5 remains being secreted, which shows that the immune anergy is specific and these cytokines participate in various immune processes. The differences observed before oral treatment with OVA are strongly correlated with mice response after being fed and further immunized with antigen.

## Author Contributions

SdO, FF, AdM, and AN: Experimental design, mice work, immunological analyses, statistical analysis, and paper writing. FdF: Experimental design, mice work, LF, EC, and AA: Experimental design and paper writing.

## Conflict of Interest Statement

The authors declare that the research was conducted in the absence of any commercial or financial relationships that could be construed as a potential conflict of interest.
